# *Trans*-Homophilic Interaction of CADM1 Activates PI3K by Forming a Complex with MAGuK-Family Proteins MPP3 and Dlg

**DOI:** 10.1371/journal.pone.0082894

**Published:** 2014-02-04

**Authors:** Shigefumi Murakami, Mika Sakurai-Yageta, Tomoko Maruyama, Yoshinori Murakami

**Affiliations:** Division of Molecular Pathology, Institute of Medical Science, The University of Tokyo, Tokyo, Japan; II Università di Napoli, Italy

## Abstract

CADM1 (Cell adhesion molecule 1), a cell adhesion molecule belonging to the immunoglobulin superfamily, is involved in cell-cell interaction and the formation and maintenance of epithelial structure. Expression of CADM1 is frequently down-regulated in various tumors derived from epithelial cells. However, the intracellular signaling pathways activated by CADM1-mediated cell adhesion remain unknown. Here, we established a cell-based spreading assay to analyze the signaling pathway specifically activated by the *trans*-homophilic interaction of CADM1. In the assay, MDCK cells expressing exogenous CADM1 were incubated on the glass coated with a recombinant extracellular fragment of CADM1, and the degree of cell spreading was quantified by measuring their surface area. Assay screening of 104 chemical inhibitors with known functions revealed that LY294002, an inhibitor of phosphoinositide 3-kinase (PI3K), efficiently suppressed cell spreading in a dose-dependent manner. Inhibitors of Akt and Rac1, downstream effectors of PI3K, also partially suppressed cell spreading, while the addition of both inhibitors blocked cell spreading to the same extent as did LY294002. Furthermore, MPP3 and Dlg, membrane-associated guanylate kinase homologs (MAGuK) proteins, connect CADM1 with p85 of PI3K by forming a multi-protein complex at the periphery of cells. These results suggest that *trans*-homophilic interaction mediated by CADM1 activates the PI3K pathway to reorganize the actin cytoskeleton and form epithelial cell structure.

## Introduction

Cell surface proteins are important for recognizing the external environment and transmitting the information to the cytoplasmic regions through intracellular signaling pathways. Cell responses, such as proliferation, differentiation, apoptosis, or migration, are determined by different signaling pathways. Recently, cell adhesion molecules (CAMs)’ role in signal transduction has emerged in addition to their classical roles in cell adhesions [1], [2]. CAMs interact with growth factor receptors on plasma membranes or adaptor molecules in juxtamembrane regions. For instance, N-cadherin and NCAM interact with FGFR to promote its signaling in neuronal cells [3]. In epithelial cells, E-cadherin recruits β-catenin in its cytoplasmic domain to organize cell adhesion machinery. However, once intracellular adhesion by E-cadherin is abrogated, E-cadherin and β-catenin dissociate from each other, and β-catenin acts as an important effector in the Wnt signaling pathway; free β-catenin accumulates in the cytoplasm, moves into the nucleus, and then stimulates the transactivation of TCF/LEF for cell proliferation [4].

Cell adhesion molecule 1 (CADM1), cell adhesion molecules of the immunoglobulin superfamily (IgCAMs), contains three extracellular Ig-like loops, a single transmembrane domain, and a short intracellular carboxy-terminal tail [5]. CADM1 is also known as TSLC1, Necl-2, IgSF4A, and SynCAM1 [6]. CADM1 is expressed diffusely in the lateral membrane of cell-cell attachment sites in polarized epithelia, whereas, expression of CADM1 is frequently lost or reduced in a variety of advanced-stage human cancers of the lung, prostate, liver, pancreas, and breast [6]. Considering that the disruption of cell-cell adhesion in epithelial cells triggers tumor cell invasion and metastasis, CADM1 is one of the crucial tumor suppressors involved in cell adhesion like E-cadherin. In fact, CADM1 has a cell aggregation activity when introduced into MDCK cells lacking endogenous CADM1 expression. However, the cytoplasmic signaling pathways triggered by *trans*-homophilic interaction of CADM1 have not been fully elucidated.

The cytoplasmic domain of CADM1 in 46 amino acids contains a protein 4.1-binding motif and a class II PSD95/Dlg/ZO-1 (PDZ)-binding motif. We have demonstrated that CADM1 is connected to the actin cytoskeleton through direct interaction with protein 4.1B [7]. CADM1 also associates with members of a group of scaffolding proteins, membrane-associated guanylate kinase homologs (MAGuKs), including MPP1-3, CASK, and Pals-2, through a class II PDZ-binding motif [8]–[11]. MAGuKs contain multiple protein-protein interaction modules, including PDZ, SH3, and GuK domains, that allow the clustering of transmembrane proteins and MAGuKs themselves [12]. In neuronal synapses, many MAGuKs, such as PSD-95, SAP102, SAP97/hDlg, and CASK, are localized at pre- and post-synaptic regions and are implicated in synaptic plasticity through the clustering of receptors [13]. In addition, one MAGuK, CARMA1, associates with PKC-θ and Bcl10 and activates NFκ-B signaling in T cells [14]. Thus, MAGuK-family proteins appeared to be important downstream molecules of CAMs, including CADM1, for intracellular signal transduction. However, the precise role of the interaction of CADM1 with MAGuKs remains to be understood.

In the present study, we established a cell-based assay to identify signaling pathways involved in cell spreading mediated by *trans*-homophilic interaction of CADM1. Distinct from a simple cell adhesion, cell spreading is a process that requires cytoplasmic signaling to generate actin reorganization mediated by *trans*-homophilic interaction of CADM1. By treating cells with 104 different chemical inhibitors with known target pathways, we identified that phosphoinositide 3-kinase (PI3K) signaling leading to actin rearrangement was essential for CADM1-mediated cell spreading. We further demonstrated that CADM1 was connected to PI3K by forming a protein complex with MPP3 and Dlg at the cell-cell contact sites.

We propose that CADM1 is implicated in transmitting cell attachment signals to actin reorganization in the cytoplasm through activating the PI3K pathway for the formation and maintenance of adhesion-based epithelial structure.

## Materials and Methods

### Expression Vectors, Cell Culture, Transfection, Antibodies, Reagents, Immunoprecipitation, Western Blotting, and Cell Aggregation Assay

These are described in detail in the Methods S1.

### Purification of Recombinant CADM1-EC-Fc

HEK293 cells stably expressing a secreted form of CADM1-EC-Fc were cultured in GIT medium for 3 days after the cells reached confluence (Wako Pure Chemical Industries, Ltd., Osaka, Japan). Then, the conditioned medium was collected, and CADM1-EC-Fc was purified using the Affi-Gel Protein A MAPS II kit (Bio-Rad) and dialyzed against phosphate-buffered saline (PBS).

### Cell Spreading Assay

Coverslips were pre-coated with 50 μg/ml of poly-L-lysine (Sigma-Aldrich) and fixed with 0.5% glutaraldehyde (Sigma-Aldrich) in 24-well plates. The glasses were then incubated with 50 nM of CADM1-EC-Fc or control mouse IgG for 10 min and blocked with 1% bovine serum albumin (BSA, Sigma-Aldrich) in Hank’s Balanced Salt Solution (HBSS) (Invitrogen). Then, MDCK cells (3×10^4^) were plated on the glasses and incubated at 37°C for 60–70 min as indicated. After incubation, cells were fixed with 4% paraformaldehyde and subjected to immunofluorescence labeling with Alexa Fluor-labeled phalloidin. Cells were imaged with an epifluorescence microscope (Zeiss), and the surface area of GFP positive cells was measured by the AutoMeasure software module, AxioVision Version 4 (Zeiss). To evaluate the activities of inhibitors, the surface area of cells with each inhibitor was normalized to that of cells on IgG with DMSO, and then the value of cells on CADM1-EC-Fc with DMSO was set as 1. More than 100 cells were counted, at least, for each assay as indicated in the legend for each Figure as reported previously [15]. Statistical differences were determined by Student’s t-test.

### Immunofluorescence Microscopy

Cells were fixed with 4% paraformaldehyde for 20 min, quenched with 50 mM of NH_4_Cl, and permeabilized with 0.1% Triton X-100 in PBS for 5 min. Cells were then blocked with 5% (w/v) fetal bovine serum in phosphate-buffered saline (PBS) and then incubated with primary and secondary antibodies sequentially with extensive washes between the incubation of different antibodies. Coverslips were then mounted with ProLong® Gold (Invitrogen), and cells were imaged with the epifluorescence microscope (Zeiss). Negative controls without primary antibodies were included in all experiments.

### Glutathione S-transferase (GST) Pull-down Assay

The GST- or His-fusion protein was expressed in Rosetta DE3 *Escherichia coli* and isolated with glutathione Sepharose 4B (GE Healthcare) or Ni-NTA Agarose (QIAGEN), respectively, according to the manufacturers’ protocols. For *in vitro* binding, the His-MPP3-N protein was incubated with GST-fusion proteins of CADM1 for 15 min at 4°C in a reaction buffer (50 mM of Tris-HCl, pH 7.4, 137 mM of NaCl, 0.1% Triton X-100, 10% glycerol, 0.5% BSA). The His-Dlg-N protein was added and incubated for 15 min, and then Glutathione Sepharose beads were added and further incubated for 1 h at 4°C. Beads were washed with reaction buffer and subjected to SDS-PAGE and Western blotting with anti-His antibodies. GST fusion proteins were detected by staining with Coomassie Brilliant Blue (CBB).

## Results

### Recombinant Extracellular Domain of CADM1 Mimics *Trans*-Homophilic Interaction of CADM1 and Induces Cell Spreading

**Figure 1:**
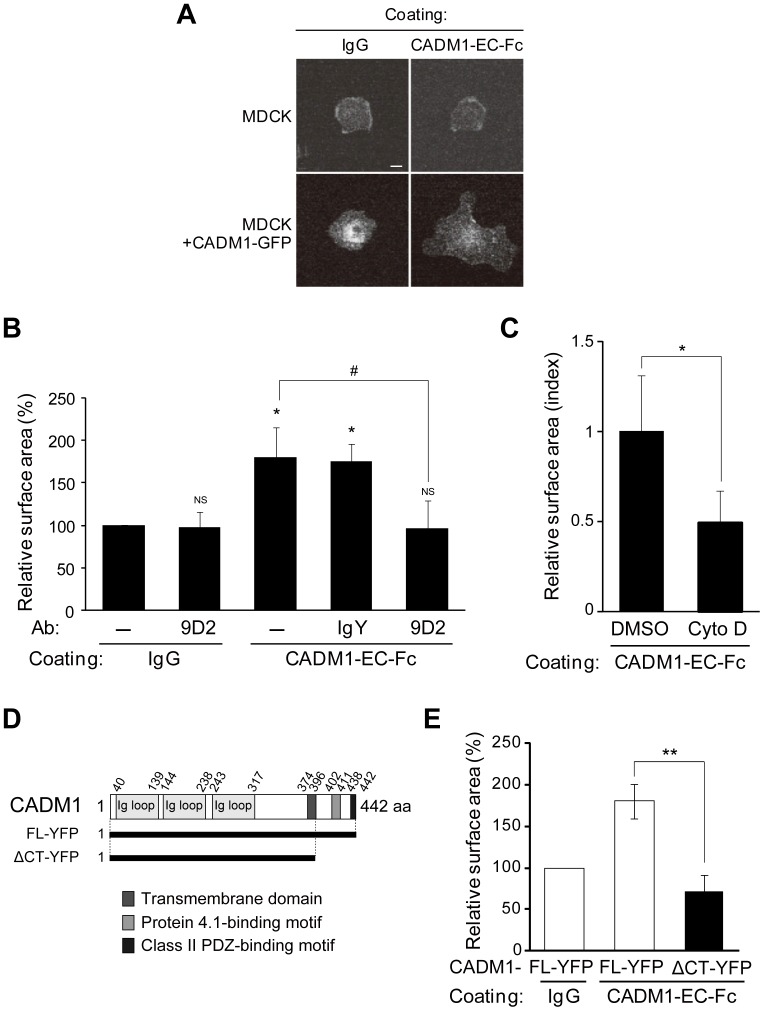
Recombinant extracellular domain of CADM1 mimics the *trans*-homophilic interaction of CADM1 and induces cell spreading. Parental MDCK cells or MDCK cells stably expressing CADM1-GFP (MDCK+CADM1-GFP) were incubated on coverslips coated with control IgG or recombinant proteins consisting of the extracellular fragment of CADM1 fused to Fc fragments of mouse IgG (CADM1-EC-Fc). After 60 min, the cells were visualized by staining the actin cytoskeleton with Alexa Fluor 568-labeled phalloidin. (A) Representative images of F-actin in cells incubated on control IgG- or CADM1-EC-Fc-coated glasses as indicated. Bars: 20 μm. (B) MDCK+CADM1-GFP cells were incubated on IgG or CADM1-EC-Fc in the presence or absence of control human IgG or anti-CADM1 antibody, 9D2 (10 μg/ml). Cell spreading was quantified by measuring the average surface area of cells. Relative value to cells on the IgG-coated glass without antibodies was shown. More than 100 cells were counted in the assay. *, p<0.05; NS, no significant difference (vs. cells on IgG without antibodies). #, p<0.05. (C) Cell spreading assay using MDCK+CADM1-GFP cells incubated on IgG or CADM1-EC-Fc with DMSO or 1 μM of Cytochalasin D (Cyto D). The area was normalized to that of cells on IgG with DMSO, and the relative value to cells on CADM1-EC-Fc with DMSO was shown. More than 180 cells were counted in the assay. *, p<0.05. (D) A schematic representation of CADM1 protein structure. The YFP-fusion proteins of full-length CADM1 (CADM1-FL) and its deletion mutant lacking the cytoplasmic fragment (CADM1-ΔCT) were shown. (E) Cell spreading assay of MDCK cells stably expressing CADM1-YFP-FL or CADM1-YFP-ΔCT that were incubated on IgG or CADM1-EC-Fc. Relative value of cell surface area to that of CADM1-YFP-FL cells on IgG-coated glass was shown. More than 230 cells were counted in the assay. **; p<0.01. (B, C, and E) The results presented are mean ± SD of three independent experiments.

We first established this cell spreading assay to identify the signaling pathway specifically activated by *trans*-homophilic interaction of CADM1 mediated by intercellular adhesion. MDCK cells stably expressing CADM1-GFP (MDCK+CADM1-GFP) or parental MDCK cells were incubated on the glass coated either with mouse IgG or with the recombinant CADM1-EC-Fc protein consisting of the extracellular fragment domain of CADM1 fused to the Fc region of mouse IgG. The following immunofluorescence staining of the actin cytoskeleton revealed that MDCK+CADM1-GFP cells showed large spread morphology when incubated on CADM1-EC-Fc-coated glass. By contrast, parental MDCK cells incubated on IgG- or CADM1-EC-Fc-coated glass or MDCK+CADM1-GFP cells incubated on IgG-coated glass showed round but not spread morphology (Fig. 1A and S1A). When the surface of the cells was measured quantitatively, the average surface area of MDCK+CADM1-GFP cells on CADM1-EC-Fc was 1.8-fold larger than that of the same cells on control IgG, although the size of the surface area was varied in each cell (Fig. 1B). To confirm that the spreading of the cells observed is mediated by *trans*-homophilic interaction of CADM1, the same assay was performed in the presence of the anti-CADM1 antibody, 9D2, which was shown to act as a blocking antibody [16]. As shown in Fig. S1B and 1B, the surface area of MDCK+CADM1-GFP cells incubated on CADM1-EC-Fc decreased significantlywhen incubated with the 9D2 antibody, whereas the area was not changed with control IgY. Moreover, the surface area of MDCK+CADM1-GFP cells was not changed by 9D2 when incubated on IgG. These results suggest that cell spreading in this assay was specifically induced by the *trans*-homophilic interaction of CADM1. Since the actin cytoskeleton is one of the main determinants of cell shape, we then investigated the effect of Cytochalasin D, an inhibitor of actin polymerization, on CADM1-mediated cell spreading. As shown in Fig. S1C and 1C, spreading of MDCK+CADM1-GFP cells on CADM1-EC-Fc-coated glass was abrogated by Cytochalasin D, but not by control DMSO. These findings suggest that the *trans*-homophilic interaction of CADM1 induces cell spreading through reorganization of the actin cytoskeleton.

Next, we investigated whether the cytoplasmic domain of CADM1 is responsible for cell spreading. To examine this, MDCK cells were stably transfected with an expression vector of a truncated form of CADM1 lacking its cytoplasmic domain that was fused to YFP (ΔCT-YFP) and subjected to cell spreading assay (Fig. 1D). It should be noted that CADM1-ΔCT-YFP was localized at cell-cell contact sites similarly to full-length CADM1 tagged with YFP (FL-YFP) in confluent MDCK cells (Fig. S1D). The surface area of MDCK cells expressing CADM1-ΔCT-YFP is significantly smaller than that of CADM1-FL-YFP cells and not different from that of MDCK cells with CADM1-ΔCT-YFP incubated on control IgG (Fig. S1E and 1E). These findings suggest that the cytoplasmic domain of CADM1, and its cytoplasmic binding proteins as well, is essential for activating signaling for the actin reorganization to lead to cell spreading.

### The PI3K Inhibitor Suppresses the Cell Spreading Mediated by *Trans*-homophilic Interaction of CADM1

**Figure 2:**
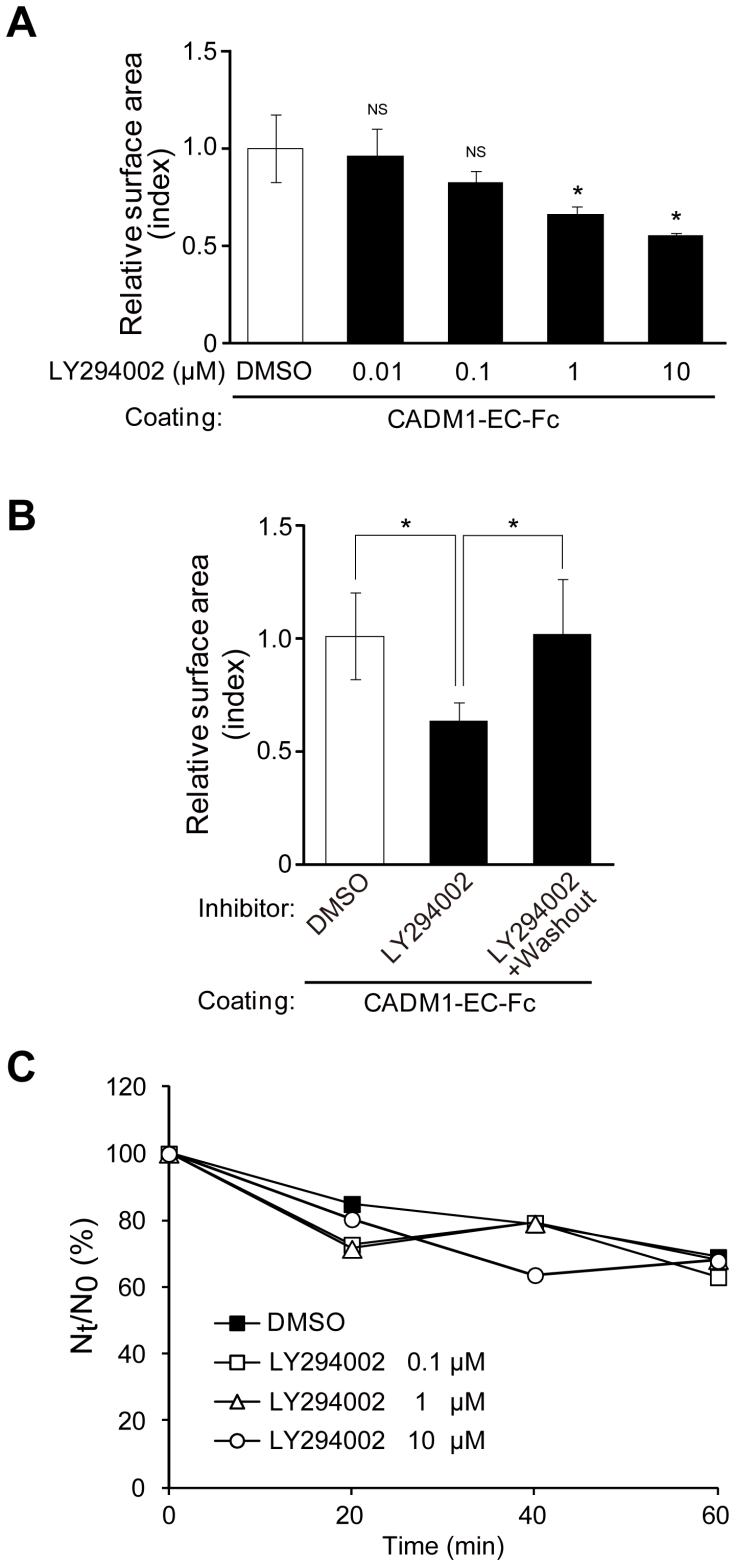
Two distinct inhibitors of PI3K suppress cell spreading mediated by *trans*-homophilic interaction of CADM1. (A) Cell spreading assay of MDCK+CADM1-GFP cells incubated on IgG or CADM1-EC-Fc in the presence of an inhibitor of PI3K, LY294002, from concentrations of 0.01 μM to 10 μM as indicated. *, p<0.05; NS, no significant difference (vs. cells on CADM1-EC-Fc with DMSO). (B) Cell spreading assay was performed using MDCK+CADM1-GFP cells cultured on CADM1-EC-Fc with 1 μM of LY294002 for 45 min and then washed and further incubated for 45 min in the presence of the same concentration of LY294002 (LY294002) or DMSO (LY294002+washout). Representative images of CADM1-GFP are shown at the top of each bar graph. The area was normalized to that of cells on IgG with DMSO, and the relative value of the cell surface area to that of cells on CADM1-EC-Fc with DMSO was shown. *, p<0.05. (A and B) The results presented are mean ± SD of three independent experiments. More than 200 and 280 cells were counted in A and B, respectively. (C) Aggregation assay of MDCK+CADM1-GFP cells in Ca^2+^- and Mg^2+^-free condition in the presence of LY294002 at the concentrations indicated. The cell aggregation was represented by the ratio of the total particle number at time *t* of incubation (Nt) to the initial particle number (N0). The data shown here indicate the average Nt/N0 in triplicate experiments.

The above findings prompted us to investigate the signaling pathway activated by CADM1-mediated cell adhesion to induce actin reorganization using cell spreading assay by treating cells with chemical compounds from the SCADS inhibitor kit (see Materials and Methods) and assessing the suppressor activity of each inhibitor in cell spreading. Among 104 chemicals we screened, two inhibitors of PI3K, LY294002 and Wortmannin, effectively suppressed cell spreading (Fig. S2A). The average cell areas treated with LY294002 and Wortmannin are 53% and 54%, respectively, in comparison with that treated with DMSO as 100%. On the other hand, the average cell surface areas treated with inhibitors of MAPK, JAK, and NF-KB in the same assay were 94%, 109%, and 96%, respectively, suggesting that the suppressor effect in cell spreading by PI3K inhibitors is significant. To confirm this inhibitory effect precisely, cells were then treated with different concentrations of LY294002, from 0.01 to 10 μM, and subjected to cell spreading assay. As shown in Fig. S2B and 2A, the surface area decreased by the treatment of LY294002 in a dose-dependent manner, where a significant difference was observed when it was treated with 1 and 10 μM of LY294002 as compared with DMSO. To exclude the possibility that LY294002 has a non-specific cytotoxic effect, cells were treated with 1 μM of LY294002 for 45 min and then washed and incubated with a fresh medium without LY294002 for an additional 45 min. Comparison of the surface area of cells revealed that cell spreading was suppressed in the presence of LY294002 for 90 min, while the suppressing effect was abrogated and the cell spreading was recovered when LY294002 was washed out (Fig. S2C and 2B), indicating that cell spreading was not irreversibly suppressed by the cytotoxicity of LY294002. Since the *trans*-homophilic interaction of CADM1 has been shown to induce cell aggregation in a suspension culture, we next examined the activity of LY294002 on CADM1-mediated cell aggregation. When cell aggregation assay was carried out, the degree of aggregate formation in cells treated with various concentrations of LY294002 did not show significant difference from that of DMSO-treated cells (Fig. 2C), showing that PI3K is not involved in intercellular adhesion activity by CADM1. These results suggest that CADM1-mediated *trans*-homophilic interaction activates PI3K to induce cell spreading but does not participate in cell aggregation activity.

### Activation of the Pathways Downstream of PI3K, Akt, and Rac1 Is Necessary for CADM1-mediated Cell Spreading

**Figure 3:**
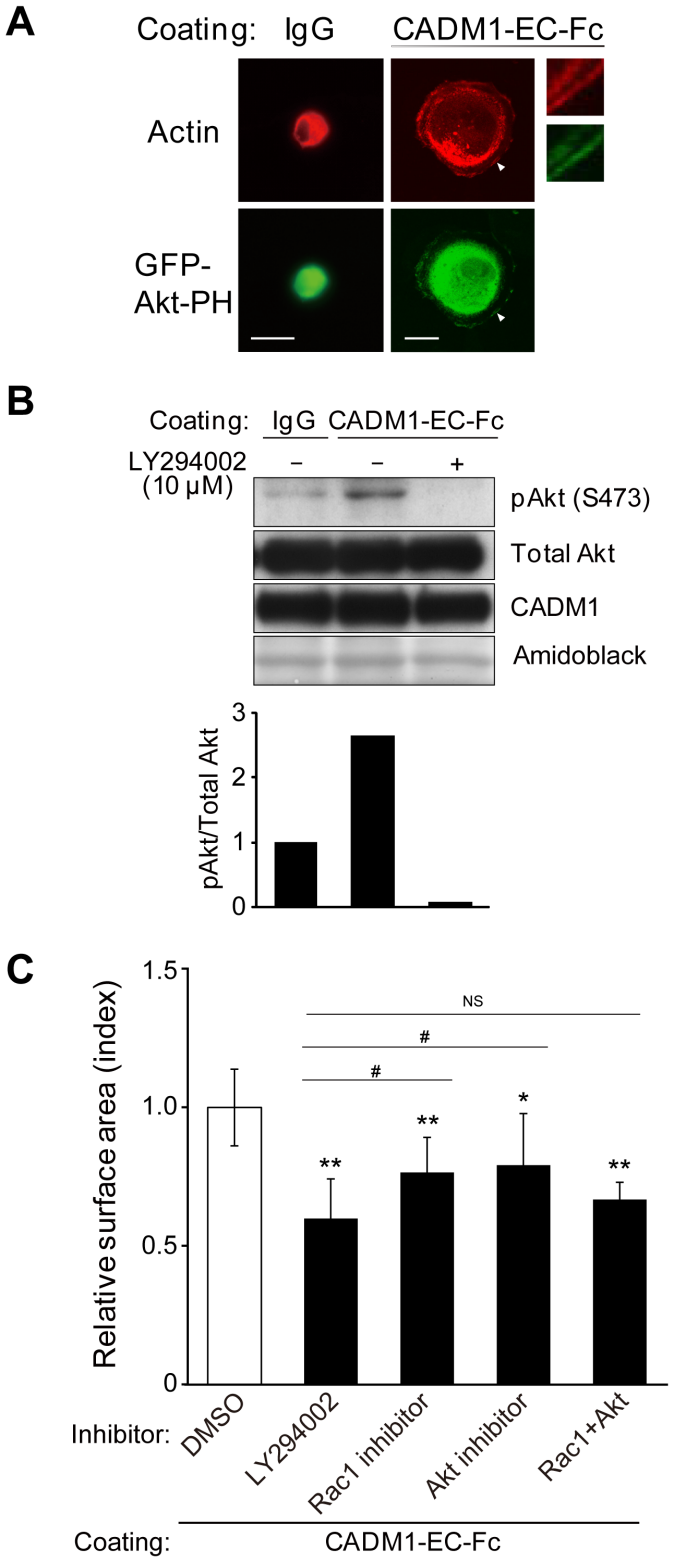
Activation of PI3K signaling is necessary for CADM1-mediated cell spreading. (A) MDCK cells stably expressing CADM1 were transiently transfected with GFP-Akt-PH and incubated on control IgG or CADM1-EC-Fc. Then, cells were visualized by staining with Alexa Fluor 568-labeled phalloidin. GFP-Akt-PH was observed at the periphery of the spreading cell where actin-rich lamellipodia were generated. High-magnification images of the region indicated by arrowhead were shown in the right panels. (B) Representative results of Western blotting analysis of phosphorylated-Akt, total Akt, and CADM1 using the lysates of MDCK+CADM1-GFP cells incubated on IgG or on CADM1-EC-Fc with DMSO (−) or with 10 μM of LY294002 (+). Note that the difference of signal intensities of Akt and p-Akt was due to the different sensitivities of antibodies and exposure time. The membrane was stained by Amido Black to confirm the equal loading of proteins. The amount of phosphorylated-Akt was normalized to that of the total Akt in each lane, and the relative value to cells on control IgG without LY294002 was calculated. The average scores of the relative values in 3 independent experiments are indicated in the lower panel. (C) MDCK+CADM1-GFP cells were incubated on control IgG or CADM1-EC-Fc in the presence of DMSO or 1 μM of the inhibitors of PI3K, Rac1 and/or Akt as indicated. The surface area was normalized to that of cells on IgG with DMSO, and the relative value to cells on CADM1-EC-Fc with DMSO was shown. The results presented are mean ± SD of five independent experiments. More than 470 cells were counted in the assay. *; p<0.05, **; p<0.01 (vs. cells on CADM1-EC-Fc with DMSO). #; p<0.05, NS; no significant difference (vs. cells on CADM1-EC-Fc with LY294002).

Then, we analyzed how PI3K was activated by CADM1-mediated cell attachment to lead cell spreading. Since PIP_3_ is a major product of PI3K signaling at the plasma membrane and specifically binds to the PH domain of Akt [17], PI3K activity can be detected by the exogenously expressed fluorescent Akt-PH in the cells. Here, to examine PI3K activation and its subcellular localization, MDCK cells expressing CADM1 without tags (MDCK+CADM1) were transiently transfected with a protein fragment of the PH domain of Akt tagged with GFP (GFP-Akt-PH) and subjected to spreading assay. After 45 min of incubation on a CADM1-EC-Fc-coated plate, strong signals of GFP-Akt-PH were detected at the leading edges of MDCK+CADM1 cells where actin-rich lamellipodia were generated, indicating that PI3K is activated at the leading edges of cells in CADM1-induced cell spreading (Fig. 3A).

We further examined the activation of Akt, a well-established downstream target of PI3K for actin remodeling, in CADM1-mediated cell spreading [18]. In Western blotting analysis, the increased intensity of the signal from phosphorylated Akt was detected in MDCK+CADM1-GFP cells cultured on the CADM1-EC-Fc-coated plate as compared with that of the cells on IgG, whereas no signal was detected when cells were treated with 10 μM of LY294002 (Fig. 3B). These results suggest that phosphorylation of Akt participates in CADM1-mediated cell spreading as a possible downstream effector of the PI3K pathway. However, when examined in the cell spreading assay, the inhibitor of Akt only partially suppressed spreading of MDCK+CADM1-GFP cells as compared with LY294002 when cultured on CADM1-EC-Fc, suggesting that some additional effectors would participate in the PI3K signaling (Fig. 3C). Then we examined Rac1, another effector of PI3K implicated in actin remodeling [19]. As shown in Fig. S2D and 3C, the Rac1 inhibitor only partially suppressed CADM1-mediated cell spreading as compared with LY294002 as the Akt-inhibitor did. However, when cells were treated with both Akt- and Rac1- inhibitors simultaneously, the surface area of MDCK+CADM1 cells decreased dramatically without any significant difference from those treated with LY294002, indicating that the Akt- and Rac1- inhibitors worked additively to suppress cell spreading. These findings indicate that both Akt and Rac1 are key effectors of PI3K when activated by CADM1-mediated cell spreading.

### CADM1 Forms a Multi-protein Complex with MPP3, Dlg, and PI3K

**Figure 4:**
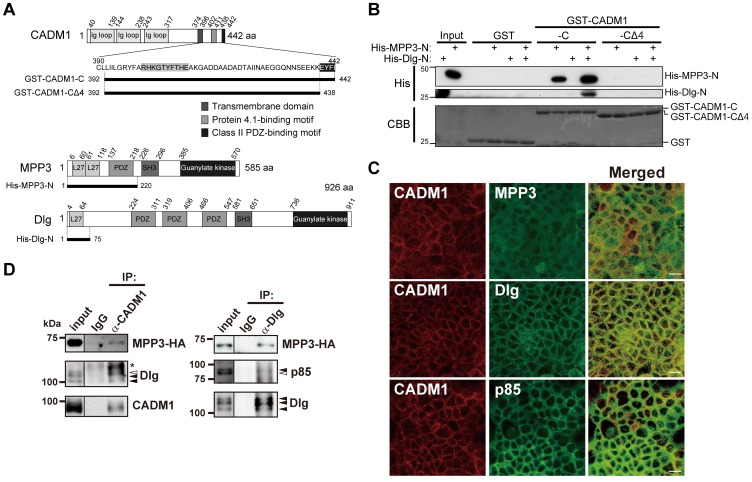
Membrane-associated guanylate kinase homologs (MAGuKs), MPP3 and Dlg, link CADM1 with p85 by forming a multi-protein complex. (A) Schematic representation of the structures of CADM1, MPP3, and Dlg proteins. In amino acid sequences of the cytoplasmic domain of CADM1, consensus sequences of 4.1- and class II PDZ-binding motifs are highlighted by grey and black, respectively. The GST-fusion protein of CADM1 with an entire cytoplasmic fragment (GST-CADM1-C) and a mutant form lacking a class II PDZ-binding motif (GST-CADM1- CΔ4) are schematically represented below. N-terminal fragments of MPP3 and Dlg were purified as His-fusion proteins and used for an *in vitro* binding assay. (B) Interaction of GST-CADM1 with His-MPP3 and/or His-Dlg was examined by GST pull-down assay. Binding proteins were detected by Western blotting using anti-His tag antibodies, whereas GST-fusion proteins were detected by staining the membrane with Coomassie Brilliant Blue (CBB). (C) Localization of CADM1, MPP3, Dlg, and p85 in confluent Caco-2 cells. Confluent Caco-2 cells were fixed and stained with anti-CADM1 antibodies (green) and anti-MPP3 (upper), anti-Dlg (middle), or anti-p85 (lower) antibodies (red). Merged images are shown in the right panel. Bars: 20 μm. (D) Lysates of Caco-2 cells expressing MPP3-HA were immunoprecipitated (IP) with control rabbit IgG and anti-CADM1 (left) or anti-Dlg (right) antibodies and analyzed by Western blotting with antibodies against HA, Dlg, p85, and CADM1, as indicated. Black arrowheads indicate signals found in both the input and immunoprecipitates, whereas white arrowheads indicate signals only found in the input. Asterisks show non-specific bands.

Finally, we analyzed possible molecules that connect CADM1 with PI3K leading to cell spreading. It has been reported that the regulatory subunit of PI3K, p85, interacts with one MAGuK, Dlg, and is recruited to cell-cell contact sites in epithelial cells [20]. Dlg further binds to another MAGuK, MPP3, which was identified as a binding partner of the cytoplasmic domain of CADM1 through their N-terminal domain [8], [21], suggesting that MPP3 and Dlg are candidates for connecting CADM1 with PI3K. To examine the possible association of these molecules, MDCK+CADM1-GFP cells were used because MDCK cells expressed significant amounts of endogenous MPP3 and Dlg proteins. We examined whether CADM1 interacts with Dlg, a key molecule in connecting with PI3K *in vitro*. For this purpose, fused proteins of GST with the cytoplasmic fragment of CADM1 (GST-CADM1-C), a derivative of GST-CADM1-C lacking C-terminal 4 amino acids corresponding to PDZ-binding motif (GST-CADM1-CΔ4), as well as His-tagged N-terminal fragments of MPP3 (His-MPP3-N) and His-tagged N-terminal fragments of Dlg (His-Dlg-N), were constructed as shown in Fig. 4A. GST pull-down assay was then performed by incubating GST-CADM1-C or GST-CADM1-CΔ4 with His-MPP3-N and/or His-Dlg-N. Western blotting analysis revealed that His-Dlg-N was recovered with GST-CADM1-C depending on the presence of His-MPP3-N (Fig. 4B). On the other hand, neither His-Dlg-N nor His-MPP3 was bound to GST or GST-CADM1-CΔ4. These results indicate that MPP3 connects CADM1 with Dlg through direct binding of the N-terminal region of MPP3 with the class II PDZ-binding motif of CADM1 and with the N-terminal region of Dlg. Next, we examined possible *in vivo* complex formation of CADM1 with MPP3, Dlg, and p85. We first demonstrated that CADM1, MPP3, Dlg, and p85 were endogenously expressed in Caco-2 cells and co-localized one another at the cell-cell contact sites (Fig. 4C). When Caco-2 cells were transiently transfected with MPP3-HA and the lysates were immunoprecipitated with anti-CADM1 antibodies, signals corresponding to MPP3-HA and Dlg were detected by Western blotting using antibodies specific to HA and Dlg, respectively (Fig. 4D, left). MPP3-HA and p85 were also co-immunoprecipitated with Dlg in the same Caco-2 lysates that expressed MPP3-HA (Fig. 4D, right). Furthermore, when endogenous CADM1 expression in Caco-2 cells was depleted by transfection of shRNA of CADM1, localization of MPP3, Dlg, and p85 at plasma membrane was almost abrogated (Fig. S3). Moreover, the spreading of cells as well as the accumulations of a protein complex of GFP-Akt-PH, MPP3, Dlg, and p85 to the periphery of spreading cells were also impaired in MDCK cells expressing CADM1-ΔCT or MDCK cells expressing wild-type CADM1 together with siDlg (Fig. S4). These results suggest that CADM1 indirectly interacts with p85 by forming a multi-protein complex with MPP3 and Dlg and participates in cell spreading.

## Discussion

CADM1 is expressed along the lateral membrane of epithelial cells and is involved in the attachment, formation, and maintenance of epithelial structure by forming a *trans*-homophilic interaction with CADM1 from adjacent epithelial cells. In the present study, to investigate signal transduction induced by CADM1-mediated intercellular adhesion, we established a cell-based assay to reconstitute an initial process of CADM1 interaction as cell spreading. The spreading of cells observed in the assay was specifically induced by *trans*-homophilic interaction of CADM1 because spreading was only observed when CADM1 was present both in the cell surface and on the glass and was abrogated by CADM1-blocking antibodies. By adding chemical inhibitors of known function in the assay and evaluating the degree of suppression in cell spreading through measuring the surface area, signaling pathways activated by *trans*-homophilic interaction of CADM1 can be identified. Among 104 inhibitors screened in the assay, two independent inhibitors of PI3K, LY294002 and Wortmannin, suppressed the cell spreading most dramatically. The suppressor activity of LY294002 in cell spreading was reversible and dose-dependent. Furthermore, the cell spreading was reversibly inhibited by a PI3K inhibitor, LY294002, in a dose-dependent manner. Additionally, by visualizing the subcellular localization of PIP_3_, a major product of PI3K, we demonstrated that PI3K was activated at the cell periphery where lamellipodia were generated. On the basis of these results, we speculate that CADM1 recruits PI3K to the cytoplasmic juxtamembrane domain to induce cell spreading. In addition to PI3K inhibitors, several known inhibitors showed partial activity in suppressing cell spreading. Some of them are related to PI3K signaling, like AKT or Rac1, while others appear to be independent of the PI3K pathways, which will be described elsewhere. MDCK cells were chosen in this assay because cell spreading was most dramatically and reproducibly observed in MDCK. In addition, we have previously demonstrated that MDCK cells transfected with a full length CADM1 showed suppressor effect in HGF-induced cell scattering in 2D-culture or tubulogenesis in 3D-culture but that transfection of CADM1ΔCT, CADM1 lacking 4.1-binding motif (CADM1Δ4.1BM), or CADM1 lacking PDZ-binding motif (CADM1ΔPDZBM) into MDCK lost the suppressor activity of scattering or tubulogenesis [27]. The functional significance of these molecules is then confirmed by transfecting shRNA of CADM1 or Dlg.

It has been demonstrated that CADM1 has potential to form heterophilic *trans*-interaction with other IgCAMs, such as CADM2/Necl-3, CADM3/Necl-1, Nectin-3, and CRTAM, depending on the types of cells [10], [22]. Therefore, analyses of *trans*-heterophilic interactions of CADM1 with other molecules using similar cell-based assay would clarify different signaling pathways activated by specific types of cell adhesion.

Since the cytoplasmic domain of CADM1 was essential for cell spreading, we analyzed the intracellular pathways leading to activation of PI3K. On the basis of the following three pieces of evidence reported so far, we hypothesized that CADM1 would recruit PI3K through MAGuKs. (1) We, and others, showed that CADM1 associates with MAGuKs, such as MPP1-3, CASK, and Pals-2, through its class II PDZ-binding domain in epithelial cells [8]–[11]. (2) The SH2 domain of p85 interacts with Dlg, one of the MAGuKs carrying class I PDZ domain, when tyrosine residues of Dlg were phosphorylated [20]. (3) Several MAGuKs, such as MPP2, MPP3, CASK, and Dlg, bind to one another through their N-terminal L27 domain [21]. In the present study, we have demonstrated that CADM1 interacts with Dlg through MPP3 at the class II PDZ-binding motif *in vitro* using GST pull-down assay (Fig. 4B). Furthermore, we have shown that MPP3 and Dlg are co-immunoprecipitated with CADM1, while MPP3 and p85 are co-immunoprecipitated with Dlg by immunoprecipitation assay *in vivo* (Fig. 4D). By fluorescence microscopy analysis, we have also confirmed that CADM1 is co-localized with MPP3, Dlg, and p85 at the cell periphery (Fig. 4), whereas the recruitment of this protein complex to the cell periphery is abrogated by depletion of CADM1 or Dlg (Fig. S3 and Fig. S4). Taken together, these 4 proteins–CADM1, MPP3, Dlg, and p85–appear to form a complex at the juxtamembrane portion at the cell periphery and play an important role in cell spreading.

Cell spreading assay also identified Akt and Rac1 as possible molecules downstream of PI3K signaling when activated by the *trans*-homophilic interaction of CADM1. Both Akt and Rac1 are molecules known to be downstream of PI3K [19], [23] in various cells, and we demonstrated that both Akt- and Rac1- inhibitors suppress spreading of MDCK cells significantly. However, quantitative measurement shows that the degree of suppression in cell spreading by Akt- or Rac1- inhibitors is only partial in comparison with that by PI3K inhibitor. On the other hand, when both an Akt- and Rac1- inhibitors are added simultaneously, cell spreading was fully suppressed. These findings demonstrate that Akt and Rac-1 act in the downstream of PI3K, but act independently of each other and that cell spreading assay has a great advantage in analyzing signaling pathways for its quantitative feature. These findings would be supported by our previous finding that Rac1 is sustained to be activated by HGF treatment when MDCK cells were transfected with full length CADM1 [26]. These findings might also be corresponding to another previous report by ours that introduction of dominant negative Rac1 suppressed lamellipodia formation of ATL cells when cultured on fibroblast, although ATL cells are not the epithelial origin [27]. Taken together, we propose a possible signaling pathway triggered by the *trans-*homophilic interaction of CADM1, as shown in Figure S5.

Expression of CADM1 is down-regulated in various carcinomas, and it is widely accepted that CADM1 is a tumor suppressor through its cell adhesive property. We have previously demonstrated that CADM1 is involved in the formation of epithelial cell structure since suppression of CADM1 expression by RNAi abrogated epithelial structure, induced simple flat morphology of cells and inhibited the maturation of cell-cell adhesion [9]. PI3K has also emerged as a major regulator of the cytoskeleton and cell polarity [24]. It is quite interesting that inhibition of PI3K by LY294002 causes a reduction in cell height but does not affect cell adhesion activity in epithelial cells [25]. Our study also indicates that the inhibition of PI3K does not affect cell adhesion activity by *trans*-interaction of CADM1 but abrogates following cell spreading activity induced by cytoskeletal remodeling. Furthermore, it has been reported that Rac1 signaling is similarly implicated in the maintenance of cell height [25] and that PI3K-Rac1 signaling serves as a key regulator for inducing the extension of cell-cell contact zones [26]. Taken together, these findings suggest that *trans*-homophilic interaction of CADM1 acts as an initial trigger on the membrane for the formation and maintenance of epithelial cell structure by activating PI3K-Rac1 pathways to reorganize the actin cytoskeleton.

In this connection, it is noteworthy that we have previously reported that overexpression of CADM1 in MDCK cells suppresses hepatocyte growth factor (HGF)-induced epithelial-mesenchymal transition (EMT), which is a well-known phenomenon associated with cancer cell invasion and metastasis [27]. In CADM1 over-expressing cells, prolonged activation of Rac1 induced by CADM1 and its cytoplasmic binding proteins appeared to inhibit EMT induction by HGF through the retention of epithelial cell adhesion. Although we did not investigate the mechanism of Rac1 activation in the study, the MAGuK-PI3K pathway could be a candidate for activating Rac1 in cells over-expressing CADM1. Thus, CADM1 appears to act as a suppressor of cancer cell invasion and metastasis by its activity in the formation and maintenance of adhesion-based epithelial cell structure.

In this study, we have demonstrated that cell-based screening assay is an effective tool for identifying low-molecular-weight compounds that target signaling pathways mediated by *tarns*-homophilic interaction of CADM1. By screening known inhibitors that suppress cell spreading, we found that the PI3K pathway was specifically activated by *trans*-homophilic CADM1 interaction. The protein complex of CADM1-MPP3-Dlg appears to recruit PI3K to the juxtamembrane region to induce actin reorganization by activating Akt and Rac1. In conclusion, the PI3K pathway is crucial for the signals mediated by *trans*-homophilic CADM1 interaction to cytoplasm, leading to cytoskeletal remodeling and the formation and maintenance of epithelial structure.

## Supporting Information

Figure S1 **Establishment of cell spreading assay.** (A) Representative images of cells analyzed by spreading assay shown in Fig. 1A. In each assay, 10 fields were imaged in duplicate and average area of cells were quantified by image J software. MDCK (a and b) and MDCK+CADM1-GFP (c and d) cells were put on IgG (a and c) or CADM1-EC-Fc (b and d), respectively, as indicated on top of images. Two fields of phalloidin-stained (a and b) and GFP (c and d) images are shown. Bars: 50 μm. (B, C, and E) Representative images of spreading assay shown in Fig. 1B (B), Fig. 1C (C), and Fig. 1E (E). Cells stained with phalloidin are shown. Bars: 50 μm. (D) Localization of CADM1-FL-YFP and -ΔCT-YFP in confluent MDCK cells. Confluent MDCK cells stably expressing CADM1-FL-YFP or -ΔCT-YFP were fixed and stained with phalloidin (red). Bars: 20 μm.
(TIF)

Figure S2 **PI3K inhibitors suppress cell spreading mediated by** ***trans*** **-homophilic interaction of CADM1.** (A) Cell spreading assay was performed with DMSO, LY294002 (1 μM), or Wortmannin (1 μM) and quantified as indicated in Fig. 1. The surface area was normalized to that of cells on IgG with DMSO, and the relative value to cells on CADM1-EC-Fc with DMSO is shown. (B, C and D) Representative images of spreading assay shown in Fig. 2A (B), Fig. 2B (C) and Fig. 3C (D). Cells stained with phalloidin are shown. Bars: 50 μm.
(TIF)

Figure S3 **Localization of MPP3, Dlg, and p85 in Caco-2 cells depleted CADM1.** (A and B) Immunofluorescence analysis of Caco-2 cells stably expressing shNegative or shCADM1_5 using antibodies indicated on top of images. Arrowheads and arrows show colocalization and mislocalization of indicated proteins, respectively, at cell-cell contact sites. Bars: 20 μm. (C) Immunoblot analysis of Caco-2 cells expressing shNegative or shCADM1_5 with anti-CADM1 and anti-GAPDH antibodies.
(TIF)

Figure S4 **Localization of GFP-Akt-PH and the components of CADM1 complex in cell spreading assay.** (A) Representative images of MDCK cells transfected with GFP-Akt-PH, CADM1-WT or ΔCT, and/or siNegative or siDlg_2 and analyzed by spreading assays indicated at the left side of images. Immunofluorescence analysis was performed using antibodies against MPP3, Dlg, CADM1, and p85 as indicated. Bars: 20 μm. (B) Immunoblot analysis of MDCK cells transiently tranefected with siNegative, siDlg_1, or siDlg_2.
(TIF)

Figure S5 **Schematic representation of the signaling pathways mediated by** ***trans*** **-homophilic interaction of CADM1 to cell spreading.** When attached on the glass coated with CADM1-EC-Fc (upper), CADM1-expressing cells activate PI3K through MPP3 and Dlg, induce actin reorganization, and show cell spreading (lower).
(TIF)

Methods S1 **Expression vectors, Cell culture and transfection, Antibodies and reagents, Immunoprecipitation and Western blotting, and Cell aggregation assay.**
(DOCX)
