# Correction: *Trans*-Homophilic Interaction of CADM1 Activates PI3K by Forming a Complex with MAGuK-Family Proteins MPP3 and Dlg

**DOI:** 10.1371/journal.pone.0110062

**Published:** 2014-09-30

**Authors:** The PLOS ONE Staff

## Notice of Republication

This article was republished on September 12th, 2014, due to Figure 3 and Figure 4 being published as duplicates of Figure S3 and Figure S4. Please download the PDF again to view the corrected article. The originally published, uncorrected article and the republished, corrected article are provided here for reference.

## Supporting Information

File S1 Originally published, uncorrected article
(PDF)

File S2 Republished, corrected article
(PDF)

## References

[1] Murakami S, Sakurai-Yageta M, Maruyama T, Murakami Y (2014) *Trans*-Homophilic Interaction of CADM1 Activates PI3K by Forming a Complex with MAGuK-Family Proteins MPP3 and Dlg. PLoS ONE 9(2): e82894 doi:10.1371/journal.pone.0082894 24503895 10.1371/journal.pone.0082894 PMC3913574

