# Supplementary figures and images for: Trans-Homophilic Interaction of CADM1 Activates PI3K by Forming a Complex with MAGuK-Family Proteins MPP3 and Dlg
Source: PLoS One. 2014 Feb 4;9(2):e82894. doi: 10.1371/journal.pone.0082894 (PMC3913574; doi:10.1371/journal.pone.0082894)

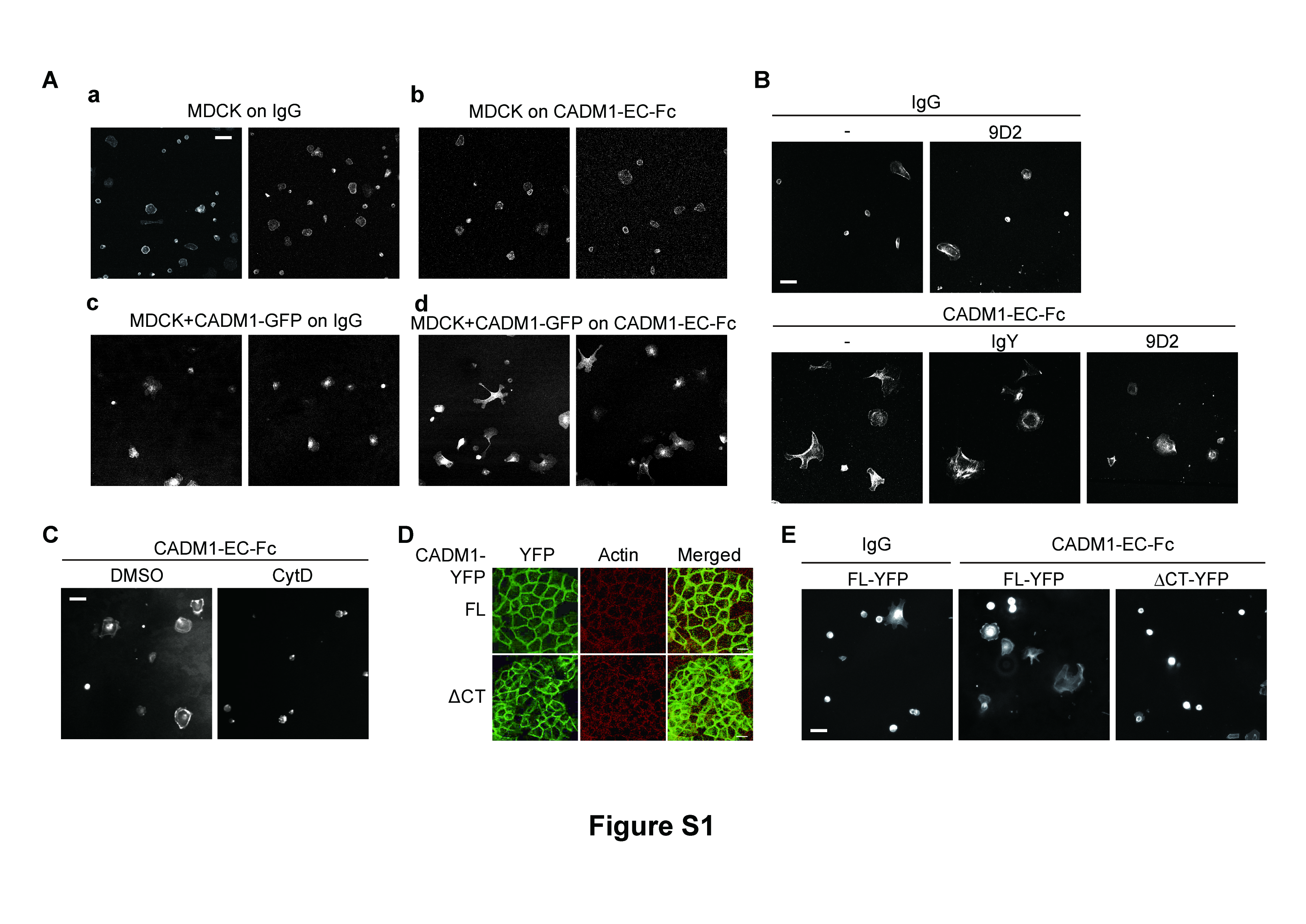

Supplement: Figure S1 — Establishment of cell spreading assay. (A) Representative images of cells analyzed by spreading assay shown in Fig. 1A. In each assay, 10 fields were imaged in duplicate and average area of cells were quantified by image J software. MDCK (a and b) and MDCK+CADM1-GFP (c and d) cells were put on IgG (a and c) or CADM1-EC-Fc (b and d), respectively, as indicated on top of images. Two fields of phalloidin-stained (a and b) and GFP (c and d) images are shown. Bars: 50 μm. (B, C, and E) Representative images of spreading assay shown in Fig. 1B (B), Fig. 1C (C), and Fig. 1E (E). Cells stained with phalloidin are shown. Bars: 50 μm. (D) Localization of CADM1-FL-YFP and -ΔCT-YFP in confluent MDCK cells. Confluent MDCK cells stably expressing CADM1-FL-YFP or -ΔCT-YFP were fixed and stained with phalloidin (red). Bars: 20 μm. (TIF) [file pone.0082894.s001.tif]

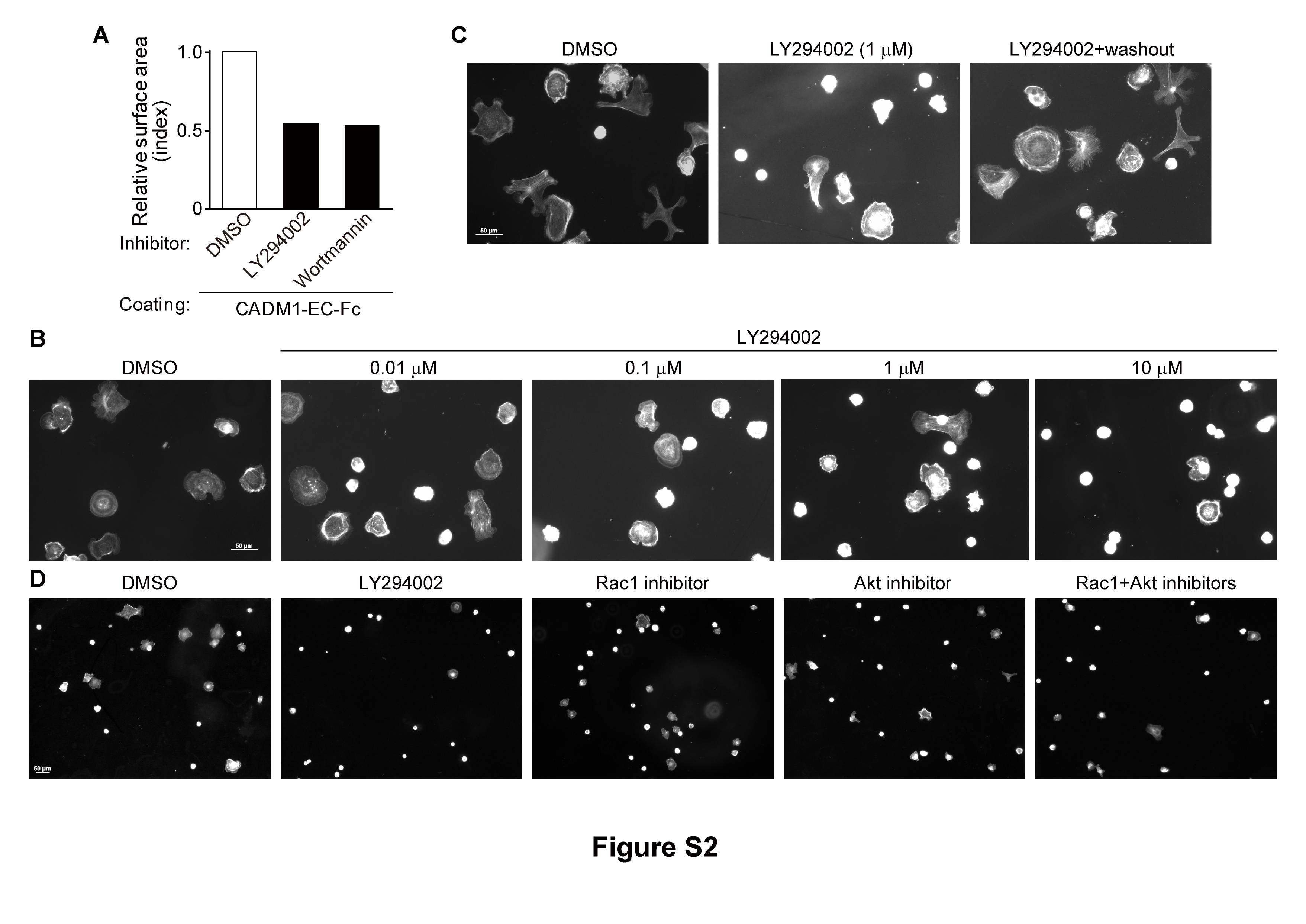

Supplement: Figure S2 — PI3K inhibitors suppress cell spreading mediated by trans -homophilic interaction of CADM1. (A) Cell spreading assay was performed with DMSO, LY294002 (1 μM), or Wortmannin (1 μM) and quantified as indicated in Fig. 1. The surface area was normalized to that of cells on IgG with DMSO, and the relative value to cells on CADM1-EC-Fc with DMSO is shown. (B, C and D) Representative images of spreading assay shown in Fig. 2A (B), Fig. 2B (C) and Fig. 3C (D). Cells stained with phalloidin are shown. Bars: 50 μm. (TIF) [file pone.0082894.s002.tif]

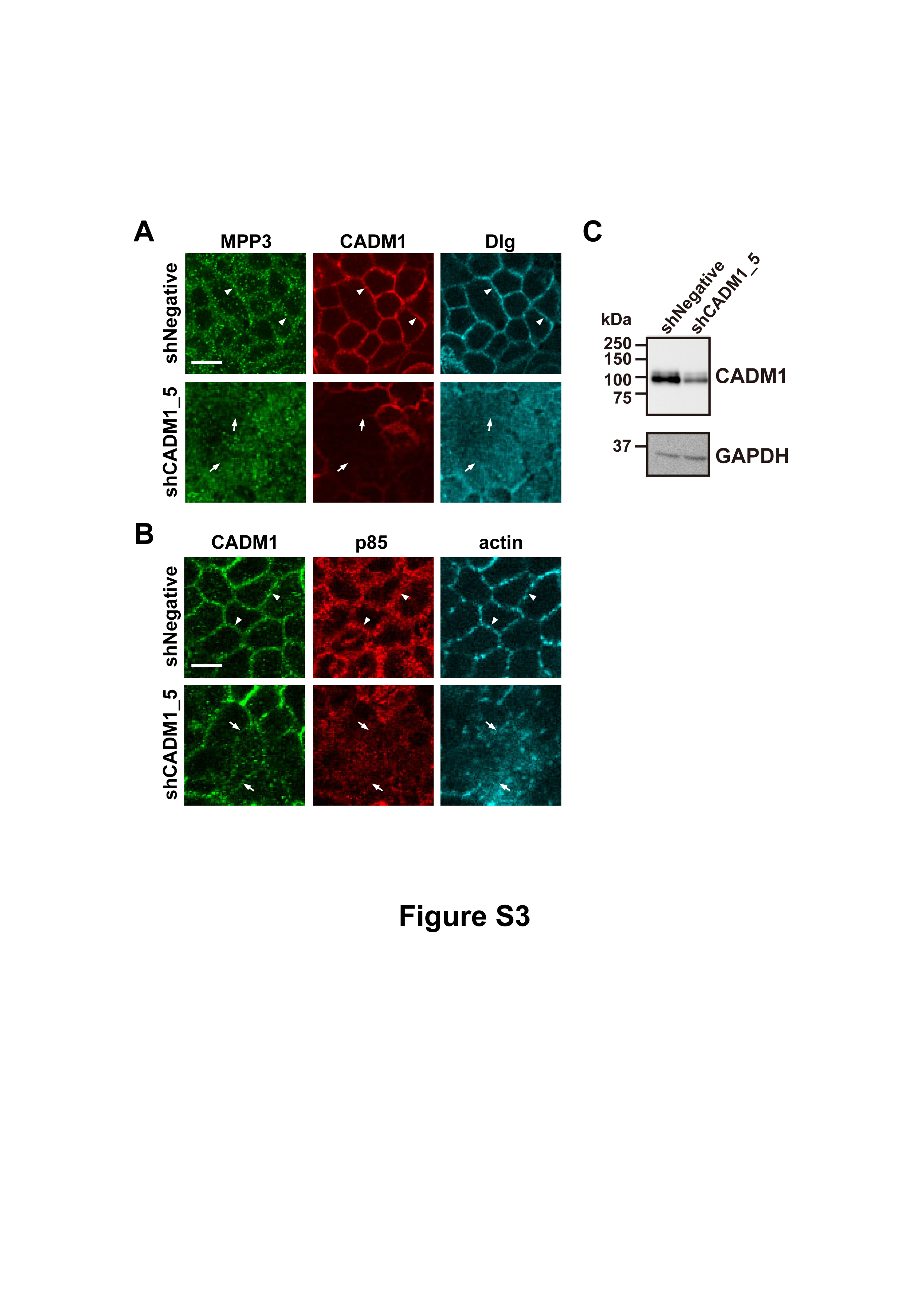

Supplement: Figure S3 — Localization of MPP3, Dlg, and p85 in Caco-2 cells depleted CADM1. (A and B) Immunofluorescence analysis of Caco-2 cells stably expressing shNegative or shCADM1_5 using antibodies indicated on top of images. Arrowheads and arrows show colocalization and mislocalization of indicated proteins, respectively, at cell-cell contact sites. Bars: 20 μm. (C) Immunoblot analysis of Caco-2 cells expressing shNegative or shCADM1_5 with anti-CADM1 and anti-GAPDH antibodies. (TIF) [file pone.0082894.s003.tif]

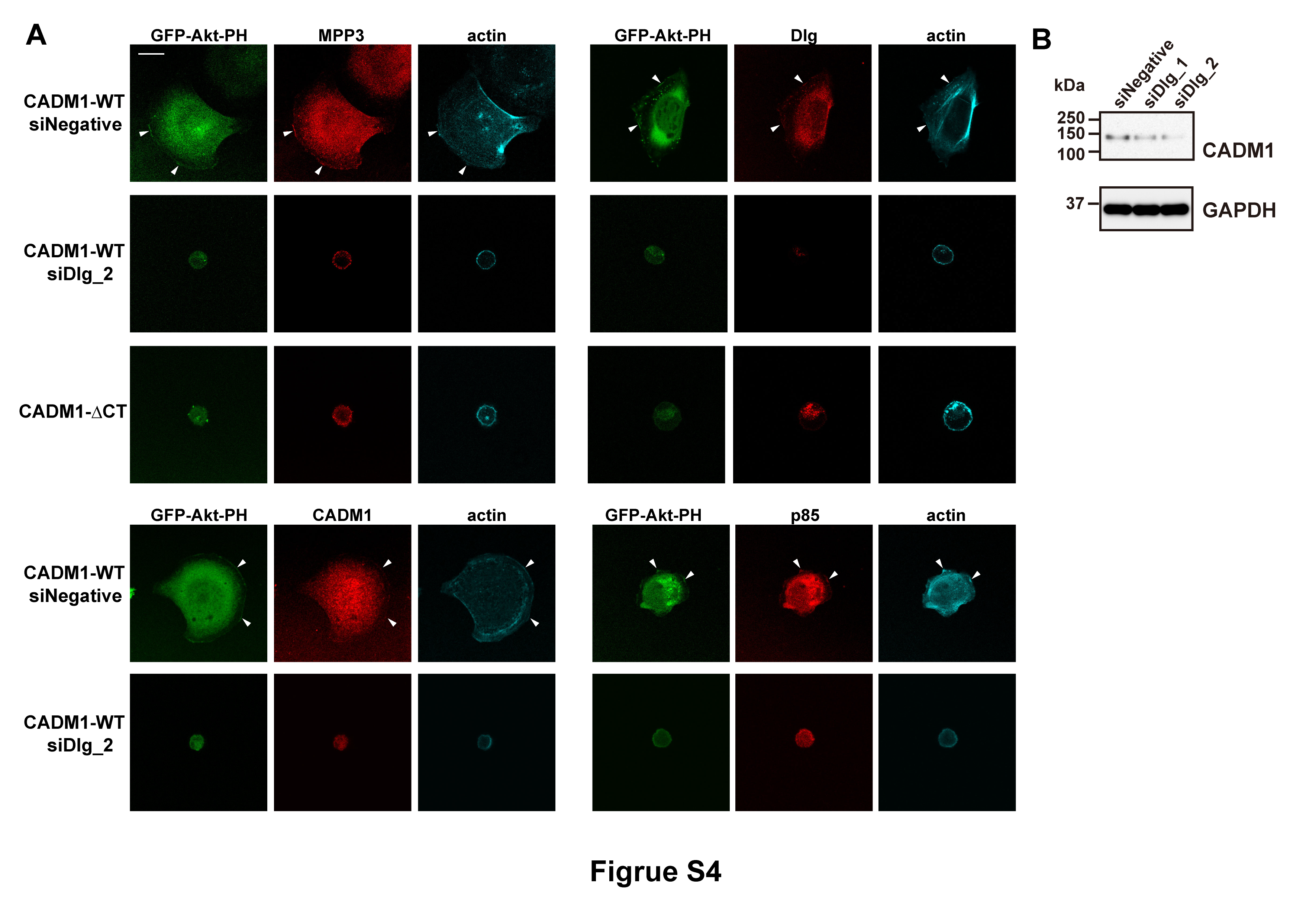

Supplement: Figure S4 — Localization of GFP-Akt-PH and the components of CADM1 complex in cell spreading assay. (A) Representative images of MDCK cells transfected with GFP-Akt-PH, CADM1-WT or ΔCT, and/or siNegative or siDlg_2 and analyzed by spreading assays indicated at the left side of images. Immunofluorescence analysis was performed using antibodies against MPP3, Dlg, CADM1, and p85 as indicated. Bars: 20 μm. (B) Immunoblot analysis of MDCK cells transiently tranefected with siNegative, siDlg_1, or siDlg_2. (TIF) [file pone.0082894.s004.tif]

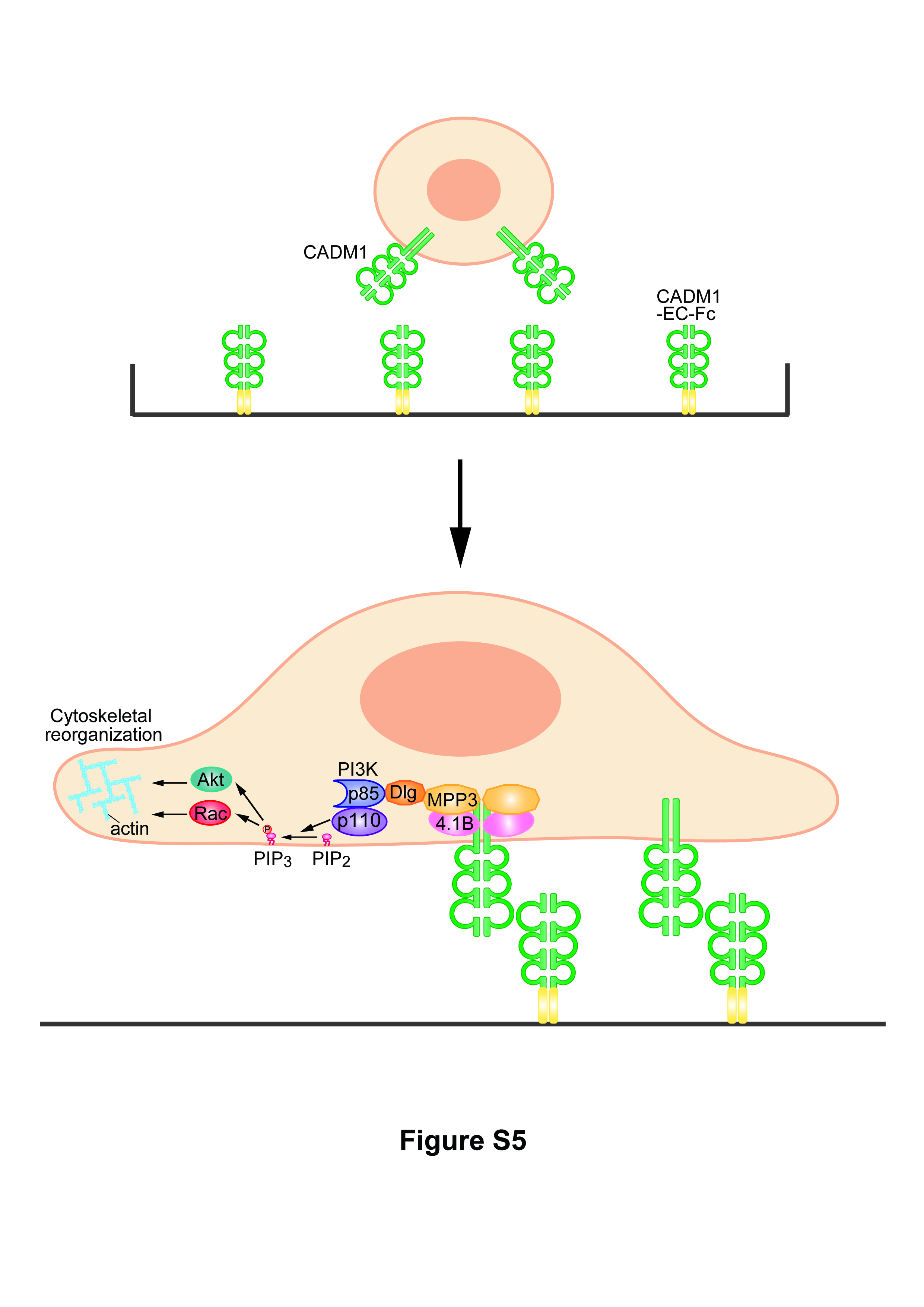

Supplement: Figure S5 — Schematic representation of the signaling pathways mediated by trans -homophilic interaction of CADM1 to cell spreading. When attached on the glass coated with CADM1-EC-Fc (upper), CADM1-expressing cells activate PI3K through MPP3 and Dlg, induce actin reorganization, and show cell spreading (lower). (TIF) [file pone.0082894.s005.tif]
