# Supplementary material for: Trans-Homophilic Interaction of CADM1 Activates PI3K by Forming a Complex with MAGuK-Family Proteins MPP3 and Dlg
Source: PLoS One. 2014 Feb 4;9(2):e82894. doi: 10.1371/journal.pone.0082894 (PMC3913574; doi:10.1371/journal.pone.0082894)
Supplement: Methods S1 — Expression vectors, Cell culture and transfection, Antibodies and reagents, Immunoprecipitation and Western blotting, and Cell aggregation assay. (DOCX) [file pone.0082894.s006.docx]

**Online Supplementary Methods**

Expression vectors

Expression vectors of CADM1, CADM1-GFP, and MPP3-HA were described previously [[5](#_ENREF_5), [9](#_ENREF_9)]. To construct a CADM1-EC-Fc expression vector, a cDNA sequence encoding the extracellular domain of CADM1 (amino acids (a.a.) 1–375) was amplified by PCR and cloned into EcoRI and NotI sites of pcDNA3.1-IgG-Fc (a gift from T. Akaike, Tokyo Institute of Technology) in-frame. To generate the GFP-Akt-PH expression vector, a cDNA sequence corresponding to the portion of the PH domain of Akt (a.a. 1–108) was amplified by PCR and inserted into the XhoI and HindIII sites of the pEGFP-C3 vector (Clontech). To obtain CADM1-YFP expression vectors, YFP and full length (FL, a.a. 1–442) or truncated fragments lacking the cytoplasmic domain (ΔCT; a.a 1-396) of CADM1 cDNA were amplified by PCR and cloned in tandem into the NheI and XhoI sites of pcDNA3.1/Hygro(+) (Invitrogen). The cytoplasmic domain of CADM1 (a.a 397–442) and its truncated fragment lacking the C-terminal 4 amino acids (a.a 397–438) were amplified by PCR and cloned into the EcoRI and SalI sites of pGEX-4T-1 (GE Healthcare) to generate GST-CADM1-C and GST-CADM1-CΔ4, respectively. To construct expression vectors of His-fusion proteins, N-terminal fragments of Dlg cDNA (Dlg-N; a.a 1-75) and N-terminal fragments of MPP3 cDNA (MPP3-N; a.a 1-220) were amplified by RT-PCR and PCR, respectively, and inserted into the XhoI and BamHI or XhoI and SalI sites of the pET32a-c(+) vector (Novagen), respectively.

Cell Culture and Transfection

HEK293 and MDCK cells were obtained from the Health Science Research Resources Bank (Osaka, Japan), and Caco-2 cells were obtained from the American Type Tissue Culture Collection (ATCC; Rockville, MD, USA) and cultured as suppliers recommended. Plasmid transfection was carried out using Lipofectamine LTX (Invitrogen) or FuGENE 6 (Roche Diagnostic, Basel, Switzerland) according to the manufacturers’ instructions. To obtain stable transfectants, cells were selected in medium with 400 μg/ml of G418 for 14 days.

Antibodies and Reagents

A rabbit Anti-His (G-18) antibody used for GST pull-down and a rabbit polyclonal Anti-Dlg antibody (H-60) were purchased from Santa Cruz Biotechnology (Santa Cruz, CA). A rat monoclonal anti-HA antibody (clone 3F10) was purchased from Roche (Indianapolis, IN, USA). A rabbit polyclonal anti-p85, rabbit monoclonal anti-Akt (C67E7), and mouse monoclonal anti-phospho-Akt (S473, clone 587F11) antibodies were purchased from Cell Signaling Technology (Beverly, MA, USA). A rabbit polyclonal anti-CADM1 antibody (c-18) and a chicken monoclonal anti-CADM1 antibody (9D2) were described previously [[15](#_ENREF_15), [16](#_ENREF_16)]. A rabbit polyclonal anti-MPP3 antibody was described previously [[9](#_ENREF_9)]. Normal mouse IgG was from Sigma. Alexa Fluor 568-labeled phalloidin and Alexa Fluor 488- and Alexa Fluor-546-labeled secondary antibodies were purchased from Molecular Probes (Eugene, OR). HRP-labeled secondary antibodies were from GE Healthcare. A series of inhibitors used in the screening was obtained by a Grant-in-Aid for Scientific Research on the Priority Area “Cancer,” the Ministry of Education, Culture, Sports, Science, and Technology, Japan as a SCADS (Screening Committee of Anticancer Drugs) inhibitor kit, which is composed of 192 chemicals commonly used in cell biology. Inhibitors of Akt (1L-6-Hydroxymethyl-*chiro*-inositol 2-(R)-2-O-methyl-3-O-octadecylcarbonate), Rac1 (NSC23766), and PI3K (LY294002) were purchased from Calbiochem. Cytochalasin D was obtained from WAKO.

Immunoprecipitation and Western Blotting

Cell lysates were prepared using lysis buffer (50 mM of Tris-HCl, pH 7.5, 150 mM of NaCl, 1% Triton X-100, and 1 mM of EDTA) with protease inhibitors. SDS-PAGE and Western blotting were performed as described previously [[7](#_ENREF_7)]. Immunoprecipitation assay was performed as described elsewhere [[7](#_ENREF_7)] after cells were lysed in lysis buffer (1% Brij98, 25 mM of Hepes, pH 7.5, 150 mM of NaCl, 5 mM of MgCl_2_, 1 mM of DTT). Briefly, 1–2 mg of cell lysates was pre-cleared with protein A sepharose (GE), incubated with 1μg of rabbit polyclonal anti-CADM1 or anti-Dlg antibody for 30 min at 4 °C, and then protein A sepharose was added and incubated overnight at 4 °C. Beads were washed with lysis buffer four times, and bound proteins were eluted in SDS sample buffer.

Cell Aggregation Assay

A cell aggregation assay was performed as described previously [[17](#_ENREF_17)]. Briefly, subconfluent layers of MDCK+CADM1-GFP cells were detached by incubation in 0.2% trypsin/1mM of EDTA at 37 °C for 5 min and dispersed by gently pipetting. After washing in Ca^2+^- and Mg^2+^-free HBSS containing 5 mM of EDTA, 1.5 x 10^5^ cells were reseeded in 12-well plates pre-coated with BSA and rotated on a gyratory shaker at 37 °C for 20, 40, or 60 min in the presence of 0.1–10　μM of LY294002. Aggregation was stopped with the addition of 2% glutaraldehyde. The extent of aggregation was represented by the ratio of the total particle number at time *t* of incubation (Nt) to the initial particle number (N0).
